# Is Gut Involvement a Cause or Effect of COVID-19?

**DOI:** 10.21315/mjms2021.28.6.14

**Published:** 2021-12-22

**Authors:** Khairil Khuzaini ZULKIFLI, Phei Oon TAN, Nazri MUSTAFFA, Yoen Young CHUAH, Raman MUTHUKARUPPAN, Zheng Feei MA, Yeong Yeh LEE

**Affiliations:** 1 Faculty of Medicine, Universiti Teknologi MARA, Sungai Buloh, Selangor, Malaysia; 2 School of Medical Sciences, Universiti Sains Malaysia, Kubang Kerian, Kelantan, Malaysia; 3 Division of Gastroenterology and Hepatology, Department of Internal Medicine, Ping Tung Christian Hospital, Ping Tung, Taiwan; 4 Department of Medicine and Gastroenterology Unit, Hospital Queen Elizabeth, Kota Kinabalu, Sabah, Malaysia

**Keywords:** COVID-19, SARS-CoV-2, ACE2, gastrointestinal tract, liver

## Abstract

Digestive disorder symptoms in COVID-19 may be similar in form to post-infectious functional gastrointestinal disorder (PI-FGID). To cause clinical effects, SARS-CoV-2 must reach the bowels and gastric hypochlorhydria may facilitate such transit. Asian elderly are predisposed to greater infection rate and severity of COVID-19, and the high prevalence of gastric atrophy and intake of proton-pump inhibitor in this aged group might explain the risk. Persistence shedding of SARS-CoV-2 in stools indicates that faecal transmission should not be disregarded. Gut involvement in COVID-19 is mediated by angiotensin-converting enzyme 2 (ACE2) receptor, which serves as the entry point for SARS-CoV-2 in the small bowel. ACE2 dysregulation has an impact on the homeostasis of gut microbiota and altered inflammatory response. Liver injury is variable in COVID-19 and is likely a result of by-stander effects rather than actual viropathic process. Further research is needed to understand if gut involvement is a cause or effect of SARS-CoV-2.

## Background

In the beginning, most have perceived the first outbreak of a novel coronavirus from Wuhan, China, in December 2019 as just another flu and some remained skeptical of its impact. Later, it was named COVID-19 on 11 February 2020 and announced as a pandemic on 11 March 2020 by the World Health Organization (WHO). The infection did not spare any nation and all countries are affected the same, with some having lesser deaths probably due to early and successful lockdown or healthcare measures. Some are experiencing second or third waves. Fast forward, as of December 2021, more than 160 million persons have been infected with a reported number of deaths already passed the three-million mark (WHO COVID-19 website: www.who.int/emergencies/diseases/novel-coronavirus-2019).

Severe acute respiratory syndrome coronavirus 2 (SARS-CoV-2) virus, the causative agent of COVID-19, is a positive-sense single-stranded RNA virus Betacoronavirus genus. SARS-CoV-2 is highly similar in sequence and binding affinity to the SARS-CoV virus, causing the SARS epidemic between 2002 and 2004 (1). The aerosol is the most common mode of transmission for both viruses but faecal sources maybe another. The primary outbreak at Hong Kong’s Amoy Gardens housing estate during SARS was believed to have resulted from toilet fumes. Faecal sources were generated into aerosols and subsequently spread the virus through inhalation and contamination (2).

Gastrointestinal disorder (GID) symptoms may precede respiratory symptoms or even occur as the sole symptom in COVID-19 (3). Is post-infectious functional GID (PI-FGID) an alternative explanation to the observed GID symptoms? How does angiotensin-converting enzyme 2 (ACE2) and SARS-CoV-2 fit into the gut involvement of COVID-19? What about inflammatory bowel disease (IBD) and liver involvement — are these just by-stander effects? This article aimed to address the above questions based on the potential causes versus effects of COVID-19 on the gut.

## Clinical Characteristics of COVID-19-Associated GID Syndrome – is this PI-FGID?

Outside of the respiratory system, the clinical effects of COVID-19 in other organs remain somewhat elusive, including the Gastrointestinal (GI) tract (4). In early reports from China, GID symptoms such as diarrhoea, nausea, vomiting and abdominal pain were described as the less common features of COVID-19. Subsequent reports, however, have shown that GID symptoms were not uncommon, might precede respiratory symptoms and could be the sole presentation in some cases of COVID-19. For example, in a retrospective study by Luo et al. (5) involving 1,141 hospitalised patients, 16% had GID symptoms as their only COVID-19 feature, with diarrhoea and abdominal pain in 37% and 25% of patients, respectively. In another study, Pan et al. (6) have shown that those with digestive symptoms sought healthcare much later and as the severity of the disease worsens, the digestive symptoms were more pronounced.

The exact mechanisms for GID symptoms in COVID-19 are unknown. The natural viral enteropathic process or gut-lung crosstalk phenomenon has been speculated but not proven (6). We propose that PI-FGID may be an alternative explanation. PI-FGID is a disorder that resembles FGID, e.g. irritable bowel syndrome or functional dyspepsia but usually of acute onset following an episode of infectious gastroenteritis. Typically the causative organism is bacteria, but parasite and virus are also recognised. Our postulation is based on several observations. Firstly, both PI-FGID and COVID-19 manifested typical symptoms of diarrhoea and abdominal pain. Secondly, both had similar reported rates of digestive symptoms (prevalence of diarrhoea of approximately 10%) (7); and lastly, both shared common pathophysiology of gut dysbiosis and altered intestinal permeability. However, we do acknowledge several differences between the two disorders. First, the female gender is typically more predisposed to PI-FGID, but no gender difference was observed in COVID-19, although males tended to have a more severe disease (8). Second, while diarrhoea can occur at the onset of both disorders, COVID-19 manifested a shorter symptom duration, although more comprehensive follow-up studies are needed to determine symptom duration of COVID-19.

## How did SARS-CoV-2 Get into the GI Tract?

To cause bowel symptoms, SARS-CoV-2 must reach the bowels uninterrupted. Several studies have shown that RNA of SARS-CoV-2 was detected in saliva (9), anal/rectal swabs (10–11) and stool specimens (11–13) of COVID-19 patients. In the first reported case of COVID-19 from the United States, who had digestive symptoms, both the respiratory and stool specimens turned out positive for SARS-CoV-2 (12). However, two of the first five reported cases in Europe have positive stool samples for SARS-CoV-2 RNA despite not having any GID symptom (14). In another study involving 59 COVID-19 patients from Hong Kong, 25.4% had GID symptoms and 15.3% of them tested positive for virus RNA in stools (15). Moreover, the stool viral RNA was more likely detected in patients with diarrhoea than those without. In a subsequent meta-analysis, the pooled prevalence of stool samples positive for virus RNA was 48.1% (15).

It is unclear how SARS-CoV-2 in the aerodigestive tract could have reached the gut since, for most people with an intact gastric function, the acid in the stomach should be adequate to kill the virus. However, among the Asian elderly, where gastric atrophy is expected due to the high prevalence of *Helicobacter pylori* infection in this region, the virus could be more likely to reach the bowels. In addition to comorbidities, we speculated that gastric hypochlorhydria might offer another explanation of why COVID-19 typically affect more elderly (16) and cause more severe disease (17). There is no published evidence to support the claim but anecdotal and preliminary data exist. SARS-CoV-2 infected an 81-year-old Japanese woman who had undergone a total gastrectomy and her stools continued excreting the virus for more than two weeks (18). From our preliminary unpublished data of 106 Malaysian patients with COVID-19, the only death had occurred in a 61-year-old man without comorbidities but had reported the use of proton-pump inhibitor (PPI). The above hypothesis needs to be proven with further research.

Another finding of importance is that viral RNA detected in the gut could persist long after respiratory samples had turned negative (10–11). A study in China found that slightly more than half (53.4%) of 73 COVID-19 patients had positive stool specimens with the duration of detectable positivity of between 1 and 12 days, and 23.3% of these patients remained positive despite negative for respiratory samples (13). The argument is although the viral RNA may be detectable in stool, confirmation of viability from viral culture is lacking (19). However, subsequent detection of live SAR-CoV-2 in stool samples demonstrated that the virus is potentially viable (20). The above findings indicate that the faecal route of transmission is highly possible, and the physician should take care during lower GI procedures.

## Role of Gut ACE2 in COVID-19 – Relevance to PI-FGID and IBD

**Figure 1:**
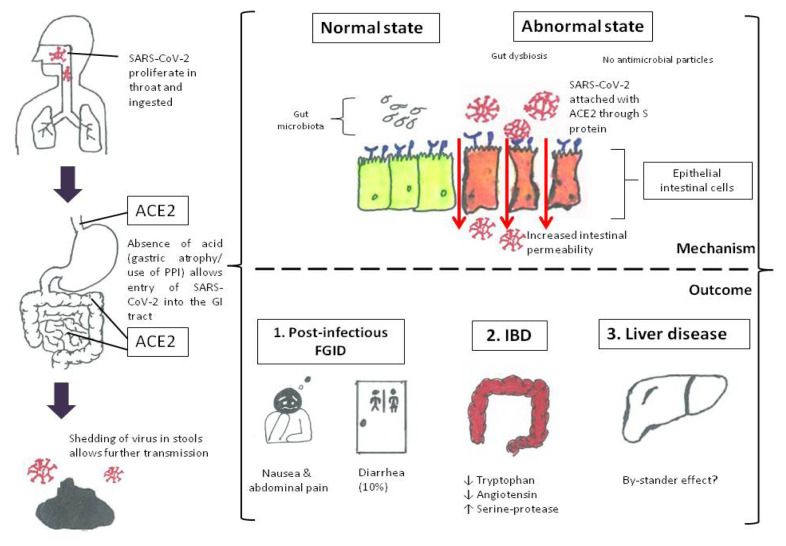
SARS-CoV-2 in the throat may transit into the GI tract unopposed in the absence of gastric acid and gets attached to ACE2 receptors or shed in stools. Although poorly understood, COVID-19 seems to affect ACE2 receptors, gut microbiota, and intestinal permeability (abnormal state). Factors including the viral load of SARS-CoV-2, affinity to ACE2 receptors and viral enteropathic properties may be important determinants of disease outcome, including FGID, IBD and liver disease (most likely by-stander or collateral effects).

Once SARS-CoV-2 enters the bowels, the virus must attach to the gut epithelium and needs to gain entry through the cellular barrier to cause digestive symptoms. The membrane protein of ACE2 is the entry receptor for SARS-CoV and SARS-CoV-2, albeit minor differences exist in several amino acid mutations at some key receptor-binding domains (21). Recent development found that transmembrane serine protease 2 (TMPRSS2), a cell surface protein expressed by epithelial cells of the respiratory and GI tract, is essential for activation (priming) of SARS-CoV-2 spike (S) protein binding to ACE2 receptor, leading to the virus’ cellular entry (22). Esophageal epithelial cells and absorptive enterocytes of the ileum and colon have an abundance of ACE2 receptors (23). Function of ACE2 in the GI tract is not exactly known. ACE2 may be essential to transport amino acid (especially tryptophan) across the epithelium and this is supported by the finding that ACE2 mutation seemed to exhibit decreased expression of antimicrobial peptides (23). Disturbed homeostasis in the gut microbial ecology may be due to changes in amino acid transport (e.g. tryptophan deficiency) in the gut. Hence, through gut ACE2, SARS-CoV-2 gains its footing in the bowels and results in dysregulation of gut microbiota and barrier function, leading to PI-FGID. If this is the case, then probiotics may work and this is also based on our work among the flood victims who developed PI-FGID (24). In the fifth version of the guideline released by China’s National Health Committee in early February 2020, probiotics have been recommended as a treatment for COVID-19 infection. Figure 1 shows the normal state of balance between gut ACE2 and microbiota and how this balance is disturbed in the presence of SARS-CoV-2.

Due to the abnormal state of balance between gut ACE2 and microbiota in COVID-19, there is a concern that IBD may worsen. In a study of 40 hospitalised patients with COVID-19, 55% reported diarrhoea, and elevated faecal calprotectin was found in patients who had ceased diarrhoea (*n* = 13/40) and also those with ongoing diarrhoea (*n* = 9/40) (25). Furthermore, the concentration of faecal calprotectin was correlated with serum interleukin-6 (IL-6) (25). The authors have suggested that the gut inflammatory response exerted by SARS-CoV-2 may potentially worsen the course of IBD (25). It is recently found that while ACE2 activities were downregulated at the tissue level in IBD, the circulating levels of ACE2 were upregulated (26). The authors have hypothesised that reduced ACE2 activity predisposes to inflammation and mural fibrosis in IBD probably through three mechanisms: i) reduced angiotensin (5–6–9–12–27); ii) tryptophan deficiency (26) and iii) elevated levels of serine-protease, the essential primer that can activate the S protein, was reportedly 10 times higher in IBD than in healthy subjects (28). In reality, there is no conclusive evidence that IBD patients are more susceptible to COVID-19. On the contrary, in an observational study of 318 IBD patients (204 ulcerative colitis and 114 Crohn’s disease), none had developed COVID-19 infection (29). In addition, several studies reported that IBD patients had had less severe COVID-19 than those without IBD (30–31). While the reason for the above observations is unknown, altered expression of ileal and colonic ACE2 receptors in IBD patients may make them less rather than more susceptible to infection (32).

## COVID-19 and Liver Disease — By-Stander Effect?

In a recent study published in the Gut journal, only 25 out of 651 patients with COVID-19 (3.8%) had pre-existing liver disease (33). Indeed, the prevalence of pre-existing liver diseases in patients with COVID-19 has ranged from 2% to 11% (34), while a large study from New York only found 0.4% of patients had pre-existing cirrhosis (35). In another large study of 1099 patients with COVID-19, only 2.1% had concomitant hepatitis B infection (36). However, these patients with existing liver diseases were more likely to report GID symptoms than patients without liver disease. It is unsure if a particular pre-existing liver condition does reduce susceptibility to COVID-19. Nevertheless, in patients with pre-existing non-alcoholic fatty liver disease (NAFLD) or steatohepatitis, it has been reported that they had more severe disease and worse outcomes, probably because of associated metabolic disorders (37).

In mild cases of COVID-19, liver injury is often transient and usually normalised without any special treatment (34). During the previous SARS epidemic, close to 60% of patients also presented with liver problems. The documented presence of SARS-CoV genetic materials in the liver tissue suggests a direct viral invasion of hepatocytes (38). In contrast, a preprint paper has reported that SARS-CoV-2 preferably bind to ACE2-expressed cholangiocytes rather than hepatocytes indicating that the variable liver injury observed in COVID-19 is probably related to cholangiocyte dysfunction rather than hepatocellular (39). However, there is an absence of significant histopathologic damage of cholangiocytes and hepatocytes in COVID-19 patients, plus bile duct parameters (e.g. alkaline phosphatase, bilirubin or gamma-glutamyltransferase) are rarely affected. It is postulated that the liver injury may result from ‘collateral or by-stander damage’ from activation of viral-induced cytotoxic T cells and the induction of a dysregulated innate immune response.

*In silico* or computerised molecular docking experiments have found high potency of directly acting antiviral (DAA) drugs on SARS-CoV-2 — sofosbuvir and ribavirin — through binding to the virus’ RdRp (RNA dependent RNA polymerase) (40). Similar virtual studies showed that telbivudine was firmly bound to the 3C-like protease (Mpro), another crucial enzyme for SARS-CoV-2 (41). Many trials are currently ongoing to repurpose drugs initially indicated for other RNA viruses to treat COVID-19, with some success (42).

## Conclusion

There are many twists in the interplay between gut and SARS-CoV-2, and further research is needed. GID symptoms in COVID-19 are not uncommon and is similar to PI-FGID in many instances. SARS-CoV-2 needs to reach the gut to cause symptoms and gastric hypochlorhydria or the use of PPI may facilitate viral transit into the bowels. Upon reaching the bowels, the ACE2 receptor allows attachment of SARS-CoV-2 but also likely explains other clinical effects in COVID-19, including IBD. On the other hand, liver involvement is likely due to the by-stander effect of COVID-19.
